# Editorial: Impact of global climate change on the interaction between plants and plant-parasitic nematodes

**DOI:** 10.3389/fpls.2023.1195970

**Published:** 2023-04-20

**Authors:** Tushar K. Dutta, Claudia S.L. Vicente, Carla M.N. Maleita, Victor Phani

**Affiliations:** ^1^ Division of Nematology, ICAR-Indian Agricultural Research Institute, New Delhi, India; ^2^ MED – Mediterranean Institute for Agriculture, Environment and Development & CHANGE – Global Change and Sustainability Institute, Institute for Advanced Studies and Research, Universidade de Évora, Évora, Portugal; ^3^ Department of Chemical Engineering, Chemical Process Engineering and Forest Products Research Centre, University of Coimbra, Coimbra, Portugal; ^4^ Department of Agricultural Entomology, College of Agriculture, Uttar Banga Krishi Viswavidyalaya, Dakshin Dinajpur, West Bengal, India

**Keywords:** plant‐parasitic nematode, climate change, nematode abundance, plant-nematode interaction, nematode management

Global climate change is an important environmental influence on plant ecosystems. Temperature, precipitation, duration and quality of sunlight, availability of nutrients, such as nitrogen, phosphorus and potassium, are determinants of plant growth that are likely to change due to global climate change effects. Climate change-induced high levels of atmospheric CO_2_ promote plant growth due to increased photosynthesis, at the cost of decreased evaporative cooling. In parallel, elevated CO_2_, temperature and altered precipitation levels strongly influence the biology of nematodes including plant and insect-parasitic nematodes. Although nematode development occur at a faster rate in warmer soil temperature, it is not yet clear about the precise implications of climate change effects on nematode biology as well as on plant-nematode interaction continuum.

Plant-parasitic nematodes (PPNs) feed on plant roots and depend on their plant hosts to survive and complete the life cycle. They are associated with nearly every economically important agricultural crop, constraining productivity. Climate change resulting from increased greenhouse gas emissions also pose a significant challenge to global crop production systems. Literature predicted that nematodes, as important ecosystem drivers, can regulate soil factors (bacterivorous, fungivorous and omnivorous nematodes improve soil health by sequestering carbon and mineralizing nitrogen) and influence a plant’s response to climate change factors, such as CO_2_ enrichment, increased temperature, erratic rainfall, and extreme weather events (storm, drought etc.). To sustain the increased crop productivity, more research must be undertaken to investigate the global climate change effects on plant-nematode interrelationship and designing of climate-smart nematode mitigation strategies.

This special issue contributes to the increased understanding of global climate change effects on plant-nematode interaction continuum and comprises a Review, and four Original Research articles.

Within this Research Topic, Dutta and Phani reviewed that according to the geo-spatial distribution of PPNs, the consequences of climate change include positive and negative effects on nematode communities. They estimated that the changing environmental factors, on one hand, will aggravate PPN damage potential by aiding to abundance, distribution, reproduction, generation, plant growth and reduced plant defense, but the phenomena including sex reversal, entering cryptobiosis, and reduced survival may act in counter direction. As the climate change effects differ according to locale and time, nematodes may react and adapt according to their location and species specificity. The bio-ecological shifts in the distribution of PPNs will require tweaking the nematode management practices towards more ‘climate-resilient’ perspectives, including effective crop scheduling, timely surveillance and monitoring of nematode distribution and spread, cover cropping, cultivation of resistant varieties, application of farmyard manure, biofumigation, etc.

Global warming has been found to cause tree mortality in the pine forests of European countries as high temperature favors the abundance of pine wood nematode (PWN) *Bursaphelenchus xylophilus*. Bacteria and fungi exist as symbionts of PWN and are closely related with pine wilt disease, although little is known about the relationship between PWN pathogenicity and associated microbiota. Cai et al. explored the relationship between microbes and PWN pathogenicity by generating a PWN-associated microbiome, revealing a significant correlation between microbial communities (bacteria and fungi) and PWN infection. The authors generated different high-throughput sequencing (HTS) datasets (transcriptome, metabolome and metagenomics) of both PWN and microbial communities.

Simulation modeling has predicted that global warming would facilitate the spread of potato cyst nematode *Globodera* spp. to newer localities making it a future invasive pest. Kud et al. investigated the belowground chemical interaction between host root and *Globodera* spp. They unraveled the molecular basis of root exudate-mediated modulation of nematode behavior. Root exudate-specific transcriptional changes in hatched pre-parasitic juveniles of *Globodera* spp. would form the basis of sustainable nematode management strategies.

Root-knot nematode *Meloidogyne incognita* cause devastating yield loss in cucumber and the damage may get severe under changing climate. Wang et al. analyzed the two major sugar metabolism processes in cucumber roots upon nematode infection. They found that raffinose family oligosaccharides (RFOs) protects the root during early nematode infection stage, although at later stage nematodes may use RFOs as their carbon source. The finding may become useful while breeding cucumber cultivars for *M. incognita* resistance by manipulating the sugar withdrawal processes.

The pest status of many organisms is likely to be altered due to global climate change, and occurrence and spread of invasive species will be high. Occurrence of invasive species causes a sharp decline in global biodiversity. The recent outbreak of terrestrial gastropod species in United States farmland has become a major concern. Schurkman et al. proposed the use of insect-parasitic nematode *Phasmarhabditis* spp. as biological control agents to tackle invasive gastropods instead of using environmentally harmful molluscicides.

Adaptative management strategies are required to control and cope with the altered pest status of parasitic nematodes, which are likely to become more threatening under changing global climate scenarios. Improved risk assessment and potential intensification of pest endurance tactics with wider inter- and intra-center collaborations will be necessary for devising new integrated nematode management modules and their disseminations. A win-win situation can be achieved regarding sustainable nematode management practices under changing climate with priority towards primary production, ecosystem services, and socio-economic feasibility of the farming practices.

## Author contributions

TD formulated the editorial article. CV, CM and VP edited the editorial article. All authors contributed to the article and approved the submitted version.

